# ATP-Dependent Diffusion Entropy and Homogeneity in Living Cells

**DOI:** 10.3390/e21100962

**Published:** 2019-10-01

**Authors:** Ishay Wohl, Eilon Sherman

**Affiliations:** Racah Institute of Physics, The Hebrew University, Jerusalem 9190401, Israel; drishaywo@walla.com

**Keywords:** image analysis, diffusion, anomalous diffusion, mechanical fluctuations, cellular entropy, cellular malignancy, liquid-liquid phase separation

## Abstract

Intracellular dynamics is highly complex, and includes diffusion of poly-dispersed objects in a non-homogeneous, out-of-equilibrium medium. Assuming non-equilibrium steady-state, we developed a framework that relates non-equilibrium fluctuations to diffusion, and generalized entropy in cells. We employed imaging of live Jurkat T cells, and showed that active cells have higher diffusion parameters (K_α_ and α) and entropy relative to the same cells after ATP depletion. K_α_ and α were related in ATP-depleted cells while this relation was not apparent in untreated cells, probably due to non-equilibrium applied work. Next we evaluated the effect of intracellular diffusion and entropy on the cell content homogeneity, which was displayed by the extent of its liquid–liquid phase separation (LLPS). Correlations between intracellular diffusion parameters, entropy and cell homogeneity could be demonstrated only in active cells while these correlations disappeared after ATP depletion. We conclude that non-equilibrium contributions to diffusivity and entropy by ATP-dependent mechanical work allow cells to control their content homogeneity and LLPS state. Such understanding may enable better intervention in extreme LLPS conditions associated with various cell malignancies and degenerative diseases.

## 1. Introduction

Intracellular dynamics is highly complex relative to thermal Brownian motion in a homogeneous viscous fluid. It includes diffusion of poly-dispersed objects in a non-homogeneous, out-of-equilibrium medium that typically demonstrates viscoelasticity, crowding, confinement and mechanical work, driven by ATP-consumption [1,2,3,4]. Such intracellular objects may include vesicles, various organelles, and cytoskeleton [5].

Intracellular viscoelasticity depends on the rheology properties of two main components: the elastic cytoskeleton, and it surrounding active gel of the crowded intracellular cytoplasm [6]. The elastic stiffness of the cellular medium is more than two-fold larger than its dissipative resistance [7]. Therefore, elasticity often dominates the shear modulus of the cellular medium. The intracellular structure has elastic properties that are non-linear and depend on the amount of load that the cytoskeleton has to resist. It also depends on generated forces from ATP-dependent myosin interactions with the actin cytoskeleton mesh [6,8,9]. Inhibition of acto-myosin interactions decreases cell elasticity by about two fold [7]. 

A living cell is in an active non-equilibrium state [10]. Therefore, its intracellular structure is expected to produce significantly more dissipation and fluctuations of forces, as compared to the same intracellular structure in equilibrium (especially a dead cell). In their comprehensive work, Guo et al. [7] described augmented, non-coherent forces in live and untreated cells in comparison to ATP-depleted cells in time scales longer than 100 ms. In shorter time scales, thermal agitation forces were dominant. According to this work, these non-equilibrium forces induce augmented intracellular diffusion with a higher power of diffusion. This effect was reversed by ATP depletion of the cells. ATP-depleted cells are assumed to experience only thermal agitation forces that drive intracellular diffusion in conditions as in thermodynamic equilibrium and with dominance of elasticity. These conditions further result in sub-diffusion characteristics with a relatively lower diffusion power [1].

Diffusion in living cells occurs in milliseconds or less and is much faster than the global dynamics of its intracellular structure, e.g., its remodeling [4,9]. Therefore, these processes can be separated in time. Assuming that the fastest processes in living cells occur in a steady-state system enables extension of thermodynamic equilibrium state functions to describe the thermodynamics properties of the cells [11,12,13]. As suggested by Oono et al. [11] and others [12,13], the concept of entropy of equilibrium thermodynamics can be extended also to non-equilibrium steady-state systems as generalized entropy. In this case, generalized entropy reflects the dispersion of microstates and energy levels of the system, which is unchanging in time. The generalized entropy is a state function on which we focus in this study. In this non-equilibrium steady-state, consumed energy (or work) and dissipation of work are key parameters that control system fluctuations. Therefore, these parameters should be considered along with the dissipation-fluctuation parameters of the system under equilibrium. While high energy consumption and high dissipation of intracellular mechanical work tends to increase fluctuations and diffusion, high viscoelasticity tends to decrease diffusion [1,6].

These considerations emphasize that living cells are not only chemical machines (with high chemical efficiency and very low dissipation), but also mechanical machines with significant energy spent on mechanical fluctuations [7].

The cell needs to balance two seemingly opposing tasks: on one hand, it needs to maintain organization of its content. Such organization may occur through compartmentalization of intracellular matter in organelles or in membraneless structures [2]. On the other hand, the cell needs to maintain fluidity (and thus, sufficient entropy) of the intracellular medium to allow its dynamic functions, including metabolism, growth and division, signaling and responses to the environment. Intracellular mechanical fluctuations could be important to control both the diffusivity and the organization of the cell content, which is closely related to its general entropy.

A very efficient way for the cell to organize its content in a dynamic and flexible way is by membraneless organelles, according to the biophysical principle of Liquid-Liquid Phase Separation (LLPS) [14]. Typical membraneless organelles are nucleoli, centrosomes, Cajal bodies, and stress granules [14]. According to the LLPS principle, liquid droplets consisting usually of a mixture of macromolecules, such as RNA and proteins, are created inside the liquid cytoplasm of living cells due to a phase separation process [15]. While creation of LLPS is controlled by a variety of parameters, including mixture composition, structure, and temperature [16], the LLPS-induced droplets may represent "active liquids" that are in a metastable state and consume ATP to control their fluidity [15].

Indeed, the cellular level of LLPS is crucial for normal cell physiology and is indicative of the cell’s balance between compartmentation and diffusivity. This level influences a wide range of important physiological steps: from DNA translation and protein synthesis to cell division [14]. The relation of LLPS to cardinal physiological processes implies also an expected relation to cellular pathophysiological states. According to recent findings, malignancy has been mainly related to a low LLPS state [17,18], while neurodegenerative diseases like amyotrophic lateral sclerosis (ALS), Alzheimer’s disease, and frontotemporal dementia (FTD) have been connected to high cellular LLPS [19,20,21,22,23].

Here, we first study the theoretical relations between cellular mechanical fluctuations, diffusivity, cellular energy levels and entropy, and then the relation of these properties to the homogeneity of cellular content, as expressed by the level of cellular LLPS. We apply our suggested theoretical framework to microscopy measurements taken on live Jurkat T lymphocytes as a model of non-adherent cells. These cells serve as an appropriate system to study cellular mechanical fluctuations and their effect on cellular content dynamics and thermodynamics. For microscopy imaging, we employed a combination of confocal and Differential Interference Contrast (DIC), which serves to capture both diffusivity and LLPS in the same cells. Our suggested framework describes the relations between multiple cellular parameters, including mechanical work, mechanical fluctuations and resulted diffusivity, entropy and content homogeneity. Specifically, we show that active cells have higher diffusion parameters ($K_{\alpha }$ and α) and entropy relative to the same cells after ATP-depletion. Correlations between intracellular diffusion parameters, entropy and cell homogeneity via an LLPS state could be demonstrated only in active cells while these correlations disappeared after ATP depletion. Therefore, it implies that the non-equilibrium contribution to diffusivity and entropy (by ATP-dependent mechanical work) is essential to enable the cell to control its content homogeneity and LLPS.

Our results and framework may enable better understanding of living cells as machines under controlled steady-state non-equilibrium conditions that utilize part of their chemical energy to produce mechanical work. This work eventually controls and balances cellular generalized entropy and homogeneity. Such a balanced control may serve as an important mechanism to adjust cell physiology. In contrast, its failure may cause major pathophysiological processes as malignancy and degenerative diseases.

## 2. Materials and Methods 

### 2.1. Materials

Complete Medium (medium): RPMI-1640, DMEM medium, heat-inactivated fetal calf serum (FCS), penicillin, streptomycin, glutamine, sodium pyruvate and HEPES obtained from Biological Industries (Kibbutz Beit Haemek, Israel). Rotenone and 2-deoxy-d-glucose from Sigma-Aldrich (St. Louis, MO, USA).

#### 2.1.1. Cells and Cloning

Jurkat (human leukemic) E6.1 (CD4^+^) T cells were a kind gift from the Samelson lab at the NIH. Jurkat cells were maintained in RPMI-1640 medium supplemented with 10% FCS, 100 U/ml penicillin, 100 μg/ml streptomycin, 2% glutamine, 2% sodium pyruvate and 2% HEPES. Cells were maintained in completely humidified air with 5% CO_2_ at 37 °C. GFP-Fibrillarin Plasmid DNA (AddGene, UK), which was used in transfections, was purified from bacterial cultures using Maxiprep columns (QIAGEN, USA). Cells were transfected with the desired DNA plasmid by using a NEON electroporator (Invitrogen).

#### 2.1.2. Sample Preparation 

For confocal imaging, coverslip preparation was as follows: coverslips (#1.5 glass chambers, iBidi) were washed with acidic ethanol at room temperature (RT) for 10 min and dried at 37 °C for 1 h. Coverslips were then incubated at RT for 15 min with 0.01% poly-L-lysine (PLL; Sigma) diluted in water. This was followed by washing and drying of the coverslips at 37 °C for 1 h. Finally, cells were suspended in imaging buffer at a concentration of 1 million and 100,000–500,000 cells were dropped onto coverslips. 

#### 2.1.3. Depletion of Intracellular ATP

Jurkat cells were washed, suspended in imaging buffer and loaded on coverslips coated with poly-L-lysine. Upon completion of measurements under normal cell condition, Rotenone 0.2 µM and 10 mM 2-deoxy-d-glucose µM were added to the imaging buffer and cells were incubated for 30 min at 5% CO_2_. At the end of incubation, measurements were performed again in the same cells.

### 2.2. Measurement System

#### Microscope

Cell were scanned using the FV-1200 confocal microscope (Olympus, Japan) equipped with an environmental incubator (temperature and CO_2_). Fluorescence imaging was performed using a 60X/1.42 oil objective, excitation at 488 nm and emission collection at 505–550 nm. Differential interference contrast (DIC) movies were taken along with fluorescence imaging. 

### 2.3. Data Analysis and Statistics

#### 2.3.1. MSD Calculations

**Figure 1:**
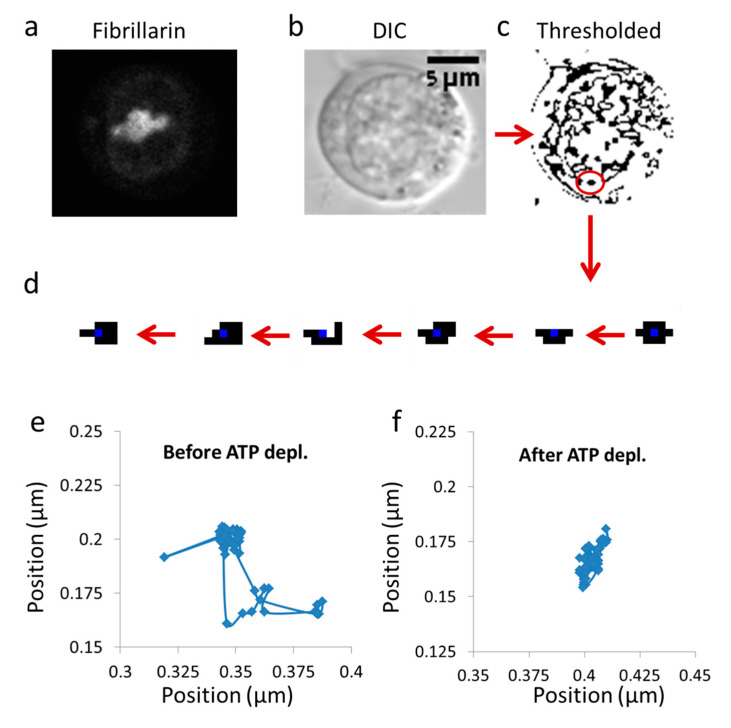
Imaging cell dynamics. (**a**) Confocal imaging of a representative live Jurkat cell, expressing fibrillarin tagged with green fluorescent protein (GFP-fibrillarin) (N = 12); (**b**) Differential Interference Contrast (DIC) image of the same cell; (**c**) Image of the cell in (**b**) after thresholding for object tracking; (**d**) An example of an individual tracked object; (**e**) Trajectories of an intracellular particle in an untreated cell (50 steps); (**f**) Trajectories of an intracellular particle in an ATP-depleted cell (50 steps).

Jurkat cells were measured using a microscope in DIC mode, utilizing × 60 magnification and conditions that were described in detail in the previous sections. The measurements included repeated measurements every 0.3 s, and analyzed over a time window of 3 s. This measurement time allowed us to effectively avoid the constrains of the limited cell size (up to ~10 µm) on diffusion. The cell image stacks were first converted to 8-bit images and thresholded (yielding binary images) to segment individual entities for tracking. Figure 1 shows an example of DIC images of a representative Jurkat cell, before and after thresholding, on which particle tracking analysis was performed.

Thresholding levels were defined according to the histogram of gray levels of the images. We noticed that a small range of thresholding values (in gray levels) were appropriate for segmentation, since too narrow threshold values caused fragmentation of the objects into isolated pixels, whereas threshold values that were too wide resulted in object contour thickening and unification. As can be seen by the size distribution of the segmented objects (see Figure S1 in Supplementary Note 1), most of these objects were in the size range of intracellular vesicles or organelles (0.1 to ~1µm). The distributions of particles diameter were similar between cells before and after ATP depletion.

Further analyses of mean square displacement (MSD) statistics and fitting (‘one term power series model fit’) were carried out using Matlab R2017b (MathWorks). Calculations of MSD values of intracellular objects were performed using the ImageJ plugin MultiTracker (The Kuhn lab; The University of Texas at Austin). 

#### 2.3.2. Data Presentation and Statistics

The acquired data was exported to Excel spreadsheets (Excel 2010, Microsoft Inc., Redmond, Washington, USA) for graph and table presentation and statistical analysis with Real Statistic Resource pack. Significance of differences between groups was calculated using Anova single factor function or Student’s t-test for paired two samples, with statistical significance set at *p* ≤ 0.05.

## 3. Results

### 3.1. Theoretical Correlation of Intracellular Diffusion to Thermodynamic Parameters of Entropy and Total Energy

We first chose to explore a possible theoretical relation between intracellular diffusivity and related generalized energy and entropy. For that, we chose the simple case of a single intracellular vesicle or element and analyzed its random motion in one dimension. The intracellular medium is considered a non-equilibrium viscoelastic medium with active (ATP-dependent) mechanical fluctuations that contribute to the particle random translocations [7]. In that crowded and heterogeneous [4,24] viscoelastic medium, time scales for non-augmented diffusion are relatively slow. For instance, the diffusion coefficient of intracellular water is 120 µm^2^/s [25], 25 µm^2^/s for GFP [26], and even slower for diffusion inside the nucleus, e.g., 0.53 µm^2^/s for GFP-fibrillarin [26] or 0.02 µm^2^/s for large intracellular particles as vesicles [7]. We further assume that the cell is in steady-state, since the diffusion process (in sub milliseconds) is much faster than global cell dynamics (estimated in seconds to minutes). When analyzing diffusion in an active viscoelastic medium, anomalous diffusion with its characteristic power law should be considered. Diffusion of molecules and particles within live cells spreads over a wide range of diffusion coefficients and between sub-diffusion and super-diffusion [24,27]. Sub-diffusion is considered when the power of diffusion is between 0 and 1, while in super-diffusion, the power of diffusion is between 1 and 2. Brownian motion occurs in an ideal homogeneous viscous medium under thermal equilibrium and has a diffusion power of 1. Factors that determine the power of intracellular diffusion relate to structure and fractal dimension [3], crowding [24], the extent of elasticity [1], and the extent of intracellular mechanical random forces [7]. Intracellular diffusion is often found to be sub-diffusive, with alpha < 1 [7].

The measured Mean Square Displacement (MSD) values of an intracellular vesicle and the corresponding time lags show a power law relation: $\langle \left(∆x^{2}\right)\rangle ≅2nK_{\alpha }∆t^{\alpha }$, where n is the number of dimensions, $K_{\alpha }$ is the diffusion coefficient, Δt is the chosen time lag and ∆x is the translocation. Repeated measurements of ∆x are typically performed and statistically analyzed to create the appropriate Probability Distribution Function (PDF) of translocations that relate to a specific time lag. While using long enough time-lags comparable to the time scale of the diffusion process, each ∆x measurement represents the composition and average result of multiple microscopic steps, through which repeated scattering of the diffusing particle occurs. The parent distribution of these microscopic steps could be Gaussian in a case of normal diffusion but may be non-Gaussian in a case of anomalous diffusion. Still, using the average results of many small steps to determine each ∆x results in a tendency of these average results to distribute normally, according to the central limit theorem (CLT).

**Figure 2:**
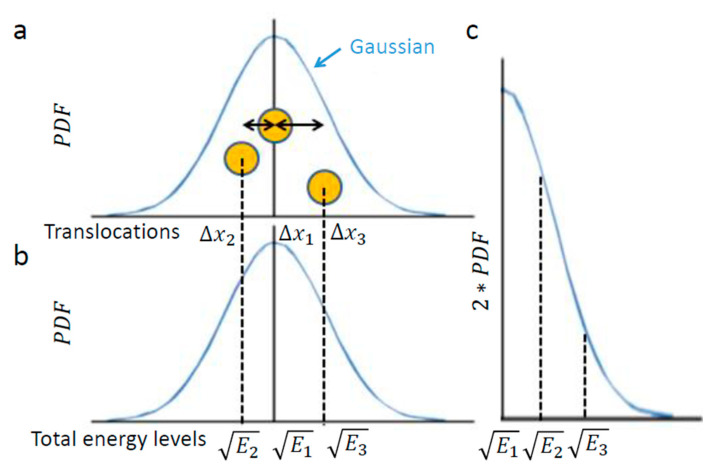
The relation of object diffusion in an elastic medium and energy levels. (**a**) Schematic representation of an intracellular element (yellow circle), like intracellular vesicle, that conducts random motion under steady-state conditions (e.g., in one dimension). Three representative positions of the element are shown, as follows: at the center of the diffusion range, at the left and at the right; (**b**) Each position corresponds linearly to the approximate square root of a specific total energy level, $\surd E_{i}$; (**c**) The probability of each total energy level can be determined and, accordingly, the generalized entropy can be approximated utilizing the calculation of entropy for the Gaussian of translocation.

The practical meaning of the above is that the PDF of translocations of the diffusing particle under these conditions approximately obeys a normal distribution, with a variance of $2nK_{\alpha }∆t^{\alpha }.$ Figure 1 illustrates this chosen example of intracellular particle (yellow circle). Based on the above section, this particle conducts diffusive motion and translocation that are analyzed over a specific time lag, upon which the distribution of its translocations ∆x around the origin (0,0) should approximate a one-dimensional Gaussian. Three representative situations are illustrated in Figure 2. In the first, the element resides in the center. In the second and third, the element diffuses from the center, either to the left or to the right, respectively.

Next, we assume that the intracellular medium is viscoelastic with an elastic component that is much more dominant [1,7] and having a low Reynolds number [28]. These assumptions imply that the intracellular element experiences potential elastic forces rather than inertia [29]. In other words, changes in energy should be attributed more to the particle’s potential than to changes in its kinetic energy. These assumptions also imply the motion of the particle under a conservative return force that is linearly related to its translocation (F=−K∆x), with $E_{potential}=0.5K{\left(∆x\right)}^{2}$. Therefore, in such a medium of negligible inertia, one may expect ∆x to reflect in a linear way the square root of the entire energy changes of the observed element.

In conclusion, the element translocations, ∆x, are in a good approximation correlated linearly to the square root of its total energy level changes. Therefore, a particle’s PDF of translocations (Figure 2a) approximates the particle’s one-tail PDF of square root of energy levels (Figure 2c). The probability of each energy level corresponds to the half-value of the corresponding probability for the appropriate translocation in each direction (Figure 2a,c)

Accordingly, the square root of energy level probabilities must be normally distributed as well. On the other hand, the differential entropy of a given Gaussian is [30]:

(1) $$Entropy_{Gaussian}=\frac{1}{2}ln\left(2\sigma^{2}\pi e\right)$$

As an extension of Shannon entropy of discrete probabilities to continuous PDFs, differential entropy is not dimensionless and is not invariant to the units that are used to measure the random variable (in our work - translocations). Due to these limitations, we mainly consider in the following analysis correlations that were derived from the differential entropy results. We overcome the lack of invariance to units by comparing results that were measured in the same way and have the same units. 

Using this approach, the measurable diffusivity (which correspond to the variance of the random Gaussian variable) was utilized to evaluate the differential entropy of translocations and energy levels. In a following step, the differential entropy was further related to the generalized entropy.

Therefore, in a case where a Gaussian describes a diffusion process (as in our case): $\sigma^{2}=2K_{\alpha }{\left(∆t\right)}^{\alpha }.$ Inserting this expression into Equation (1) produces the differential entropy of the Gaussian of translocations (i.e., the square-root of energy levels). Considering the half-Gaussian for energy levels (Figure 2c) yields the following relation to the generalized entropy:

(2) $$Entropy_{particle}\propto \frac{1}{4}ln(4\pi eK_{\alpha }\left(∆t{)}^{\alpha }\right)$$

**Figure 3:**
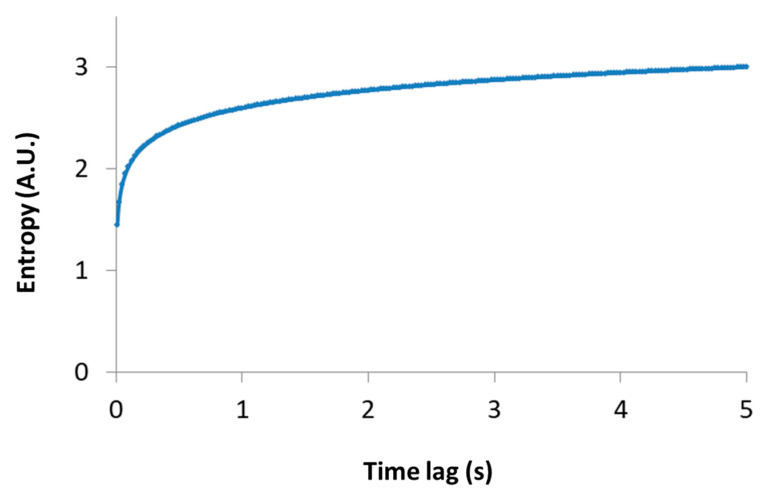
Entropy versus time lag. A schematic representation of Equation (2) is shown, where *K_α_* = 1 and α=1.

Importantly, this relation links between the thermodynamic measure of particle generalized entropy and its diffusion characteristics, namely the diffusion coefficient (*K_α_*) and the power parameter of the diffusion process (α). The time lag (∆t) must be long enough to reflect most of the possible translocation positions of the particle, and thus yields (through Equation (2)) a good approximation of the particle entropy. Note that the relation between the particle entropy and the time lag is logarithmic. Therefore, as the time-lag increases, the dependent increase in entropy becomes less significant (e.g., see Figure 3, for the case of *K_α_* = 1 and α=1). This implies that the typically limited time-lags in experiments can still provide relatively accurate estimates of the generalized entropy.

The integral of an arbitrary Gaussian function is equal to:

(3) $$\overset{\infty }{\underset{-\infty }{\int }}e^{-a{\left(x+b\right)}^{2}}\mathrm{dx}=\sqrt{\frac{\pi }{a}}$$

In the situation of diffusion motion and PDF of translocations, $a=\frac{1}{2\sigma^{2}}=\frac{1}{4K_{\alpha }{\left(∆t\right)}^{\alpha }}$. According to this relation and to Equation (3), the integral of the Gaussian is equal to:

(4) $${\mathrm{Gaussian}}_{\mathrm{integral}}=\sqrt{4\pi K_{\alpha }{\left(∆t\right)}^{\alpha }}$$

**Figure 4:**
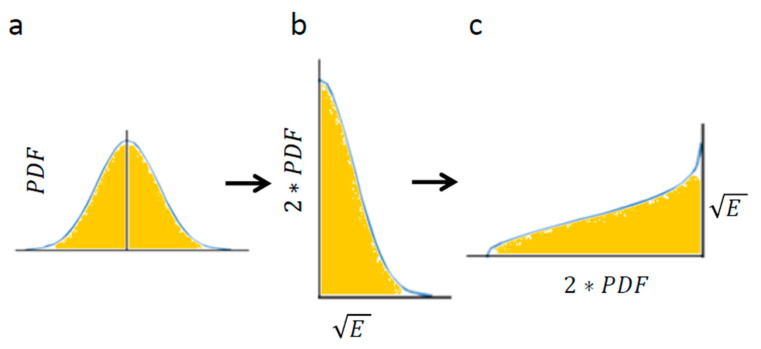
The conversion of translocations in an elastic medium into energy levels. (**a**) PDF of translocations of diffusion motion of an intracellular particle. The area below the graph is highlighted; (**b**) The corresponding graph of the linearly correlated square root of energy levels and the double values of matched probabilities. The area below the graph is linearly correlated with the area below graph (**a**); (**c**) The same as graph (**b**) except that the x and y axes are switched. The area below this graph is the same as in graph (**b**) and represents the square root of the average total energy of the particle-environment system.

Using Equation (4) enables calculation of the area below Figure 4a: the Gaussian of PDF of translocations. The Gaussian of translocations and its integral (Figure 4a) linearly relate to the Gaussian of the square root of energy levels and its integral, respectively (Figure 4b). The integral of Figure 4c has the same area as the integral of Figure 4b (because only the x and y axes were switched between these graphs).

Therefore, the result of Equation (4): $\sqrt{4\pi K\alpha {\left(∆t\right)}^{\alpha }}$ for Figure 4a is linearly correlated with the integral of Figure 4c, which in turn represents the square-root of the average total energy of the particle. Accordingly, the following relation can be determined:

(5) $$\mathrm{Average}\;\mathrm{total}\;\mathrm{energy}\propto 4\pi K_{\alpha }{\left(∆t\right)}^{\alpha }$$

It can be concluded from Equation (5) that a negative correlation between $K_{\alpha }$ and α could be defined in a case of particle diffusion in a visco-elastic intracellular medium under thermodynamic equilibrium and when the total energy is constant. This negative correlation can be expressed as follows:

(6) $$\frac{{\mathrm{Constant}}_{\mathrm{energy}}}{4\pi }∆t^{-\alpha }\propto K_{\alpha }$$

**Figure 5:**
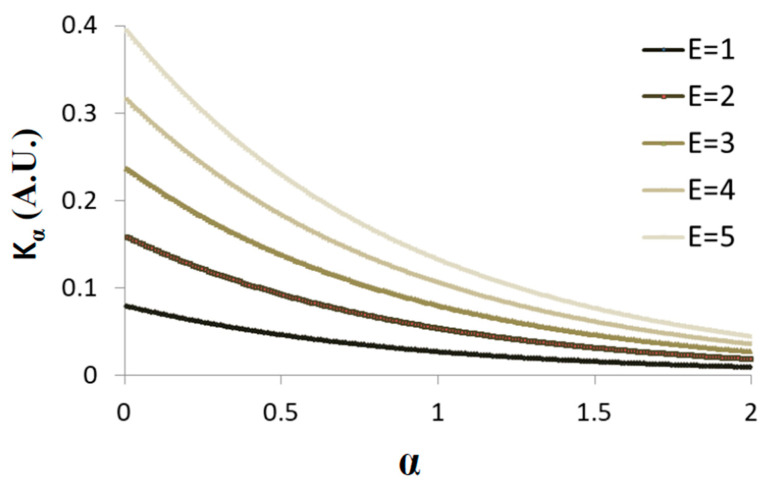
Schematic representation of Equation (5): *K_α_* versus α. The average total energy is increased from one to five (A.U.) and ∆t = 3 s.

Figure 5 presents schematically the dependence of $K_{\alpha }$ on α when the average total energy is increased from one to five (in arbitrary units) and ∆t = 3 s.

Under non-equilibrium conditions, the total energy of the system is not constant and work is done on or by the system. Under such conditions, increasing the total energy will increase both *K_α_* and α, while the ratio $\frac{∆K_{\alpha }}{∆\alpha }\;\;$ decreases (Figure 5; and the derivative of Equation (5) in respect to α, namely $\frac{\partial K_{\alpha }}{\partial \alpha }=-\frac{E∆t^{-\alpha }\ln\left(∆t\right)}{4\pi }$).

Accordingly, it is expected that under non-equilibrium conditions, when work is added to the system, both $K_{\alpha }$ and α will be larger in comparison to the same system in equilibrium; but, at the same time, the ratio $\frac{∆K_{\alpha }}{∆\alpha }$ will decrease, as work is added gradually to the system. Therefore, the gradual increase in *α* is expected to be smaller, as compared to the gradual change in $K_{\alpha }$.

Considering several similar cellular systems, all in non-equilibrium but at different energy (or work) levels, the ratio of α and $K_{\alpha }$ in each cell will be different relating to its different specific energy level and $\frac{∆K_{\alpha }}{∆\alpha }$ ratio. In this case, it will not be possible to define apparent relation between *K_α_* and α in such cellular systems.

Viñales and Desposito [31] have studied a similar model of thermally diffusing particles under applied harmonic force in a disordered-fractal environment. Based on Langevin equations analysis, they suggested that diffusion could be either restricted (sub-diffusion) or augmented (super-diffusion), depending on the relative strength of the harmonic force. In the case of augmented diffusion, *α* increases while work is added to the system. Bruno and Desposito [32] have further described an analytical framework that also utilized Langevin equations for the characterization of anomalous diffusion in living cells. They separated the fluctuating forces into two components: thermal fluctuating forces and non-equilibrium mechanical random forces. Their resultant equation for MSD showed that when there are no non-equilibrium forces, the power of intracellular diffusion has only a single component. However, when diffusion is dominated by non-equilibrium forces, the power of diffusion consists of two components: the previous equilibrium component plus a new non-equilibrium component. Relying on Bruno and Desposito theoretical results, as the system shifts from equilibrium to non-equilibrium conditions, it is possible to estimate (to a first degree) the increased *α* value in non-equilibrium as the summation of two *α* values: the basic *α* equilibrium value ($\alpha_{e}$) plus the increase in *α* due to the change from equilibrium to non-equilibrium conditions: $∆\alpha \sim \alpha_{work}$. Accordingly:

(7) $$\alpha_{non-e}\sim \alpha_{e}+\alpha_{work}\;\;\;\;$$

$\alpha_{e}$ may be related to the elasticity of the medium in equilibrium, which in turn tends to reduce *α* values. $\alpha_{work}$ relates to the work or energy that is added (in this case of active cells) to the non-equilibrium system and tends to augment the particle motion while increasing *α* in the MSD(Δt) plots.

The added mechanical work to the non-equilibrium intracellular system is expected to increase also the $K_{\alpha }$ value, as mentioned previously (Figure 5). In this way, not only the shape of the MSD(Δt) plot (i.e., *α*) is changed, but also the diffusion coefficient, $K_{\alpha }$. 

Installing Equation (7) for the combined *α* value in Equation (2) for the particle entropy yields the following relation for the combined particle entropy:

(8) $$\begin{array}{ll} {\mathrm{Entropy}}_{\mathrm{particle}}\propto & \frac{1}{4}\ln(4\pi e\alpha K_{\alpha_{non-e}}\left(∆t{)}^{\alpha_{e}+\alpha_{work}}\right)\\ & =\frac{1}{4}\ln(4\pi eK_{\alpha_{non-e}}\left(∆t{)}^{\alpha_{e}}\left(∆t{)}^{\alpha_{work}}\right)\right)\\ & =\frac{1}{4}\ln(2\pi eK_{\alpha_{non-e}}\left(∆t{)}^{\alpha_{e}}\right)+\frac{1}{4}\ln(2\pi eK_{\alpha_{non-e}}\left(∆t{)}^{\alpha_{work}}\right) \end{array}$$

Following Equation (8), the intracellular particle entropy could be considered as composed of two main components: the elasticity of the intracellular medium (first term in the summation in Equation (8)), and the mechanical work done on the intracellular medium (second term in this summation).

An adequate level of intracellular entropy is required for the proper flow of cellular metabolism and physiological flexibility. In contrast, intracellular entropy that is too high may irreversibly compromise the integrity and order of the cell, and may further lead to its malignant transformation. According to Equation (8), high elasticity of the cellular medium reduces the $\alpha_{e}$ value, and accordingly reduces the first term entropy component of the particle (the first component of Equation (8)). As a result, the total entropy is also reduced. In this way, the elasticity of the cellular medium may serve as a balancing factor that opposes the increase in entropy due to mechanical work in the cell. 

Inserting the particle energy $E_{particle}$ according to Equation (5) in the particle entropy (Equation (2)) clarifies the relation between the particle’s entropy and its energy level (up to a constant):

(9) $$\begin{array}{c} {\mathrm{Entropy}}_{\mathrm{particle}}\propto \frac{1}{4}\ln(4\pi eK_{\alpha }\left(∆t{)}^{\alpha }\right)\\ E_{particle}\propto 4\pi K_{\alpha }{\left(∆t\right)}^{\alpha }\\ {\mathrm{Entropy}}_{\mathrm{particle}}\propto \frac{1}{4}\ln\left(eE_{particle}\right)=\frac{1}{4}+\frac{1}{4}\ln\left(E_{particle}\right) \end{array}$$

**Figure 6:**
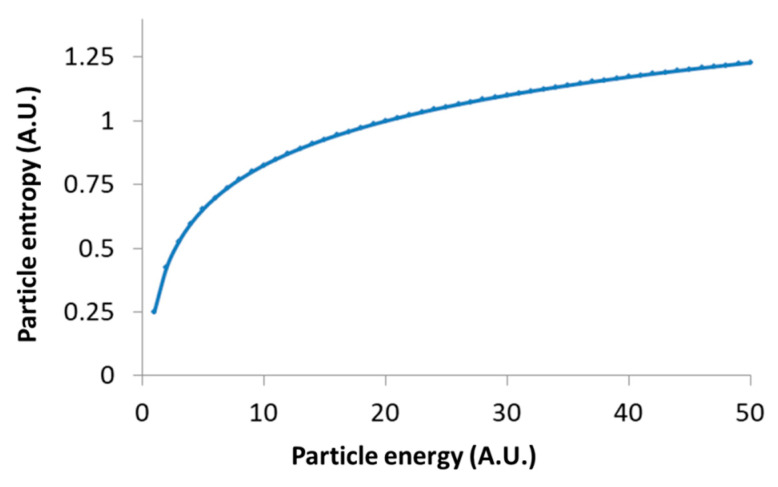
Schematic representation of Equation (9): Particle’s entropy versus the particle’s energy.

Following Equation (9), the logarithmic relation between the generalized entropy of intracellular particles and their total energy restrains the possible increase in their entropy due to increase in these particles’ energy; for instance, as a result of applied mechanical work. This logarithmic relation in Equation (9) is demonstrated in Figure 6.

In summary, for a particle diffusing in an intracellular medium, dominated by elasticity:

- The PDF of the translocations of an intracellular particle can be approximated by a normal distribution, and so does the related square root of energy levels.
- The above normal distribution of translocations, or the PDF of square root of total energy, correlates to generalized (thermodynamic) entropy of this particle system and hence, relates diffusivity characteristics to thermodynamic entropy (Equation (2)).
- The integral of the normal distribution of translocations, or the PDF of square root of total energy, correlates to the total energy of this particle system and hence leads to correlations between diffusivity parameters (*K_α_* and *α*) in thermodynamic equilibrium (Equation (6)). This correlation does not hold if energy is added to the system. Such energy is expected to increase $K_{\alpha }$ and *α* to a different extent.
- Values of *α* and of the entropy of the particle can be divided into two contributions: One due to elasticity of the medium, and the other due to the work done on the particle. The co-influence of these two contributions and the logarithmic relation between the particle’s entropy and its energy provide a mechanism for inhibiting excessive rise in cellular entropy. In turn, this may protect cells from irreversible disruption of the order of their intracellular medium.

### 3.2. Relation of $K_{\alpha }$ and α for Intracellulr Vesicle Diffusion in Jurkat T Lymphocytes

**Figure 7:**
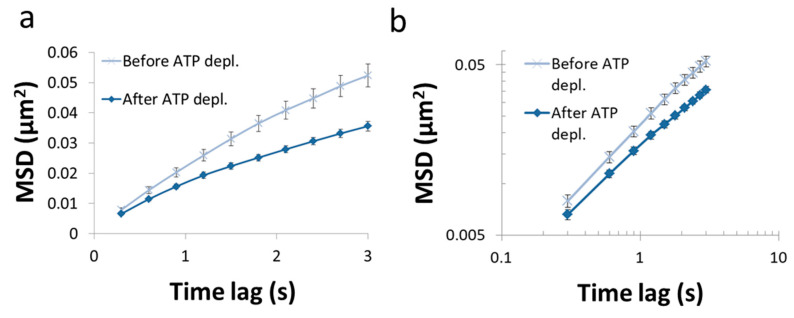
MSD vs time lag before and after ATP depletion. (**a**) Average MSD values of intracellular particles in Jurkat cells, as a function of time-lags before and after ATP depletion (N = 12). Error-bars are SEM; (**b**). The same results presented in (**a**) in logarithmic scale.

**Figure 8:**
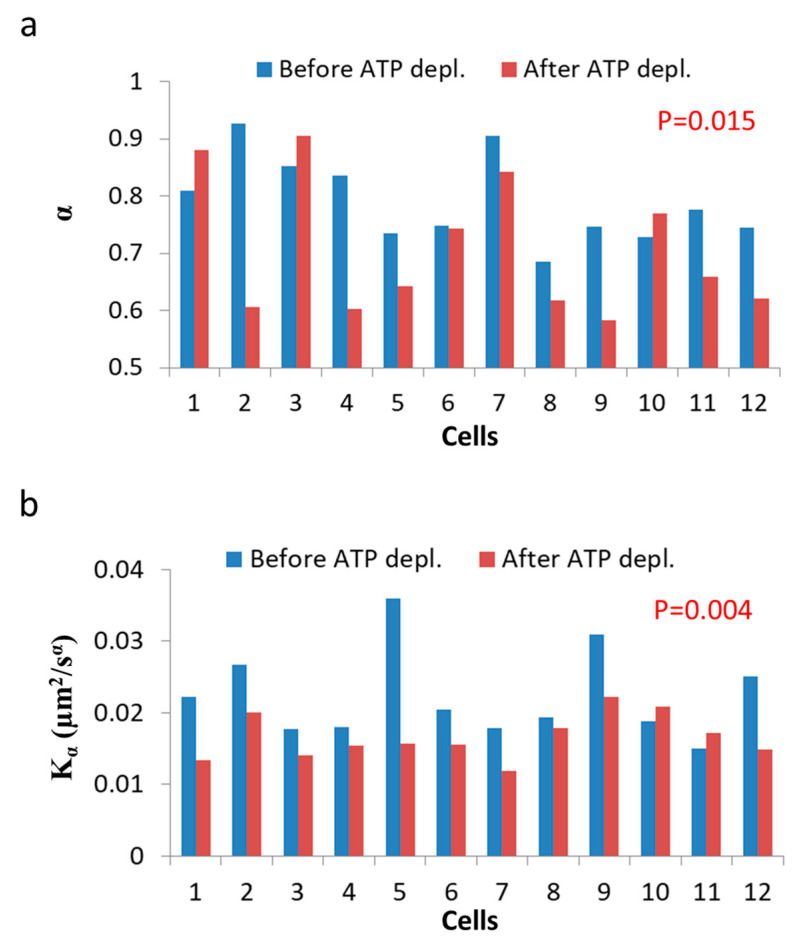
ATP depletion hinders intracellular diffusion. (**a**) *α* values for each of 12 Jurkat cells before and after cellular ATP depletion; (**b**) *K_α_* values for each of 12 Jurkat cells before and after cellular ATP depletion. *p*-values compare the results of the two cellular conditions.

In this section, we present the results of the analysis of diffusion of intracellular vesicles in 12 Jurkat T lymphocytes, each cell before and after ATP depletion. Depletion of cellular ATP was induced by 30 min incubation with 0.2 µM mitochondrial complex 1 inhibitor Rotenone together with 10mM glycolysis inhibitor 2-deoxy-D-glucose [7,33]. DIC microscopy was utilized for visualization of intracellular objects (including vesicles). For the analysis of particles’ MSD, cells were imaged repeatedly: every 0.3 s, with a total of 100 images acquired for each cell. The MSD values were calculated utilizing ImageJ plugin MultiTracker (Kuhn lab, the University of Texas at Austin) for time-lags form 0.3 s to 3 s (0.3 s gradual increase). The average MSD values were determined for that series of time-lags for each cell before and after ATP depletion (Figure 7). From these values a fit to a model of power series was conducted for each serial MSD measurement to determine the corresponding *K_α_* and *α* values for the diffusion process in each cell, before and after ATP depletion (Figure 8).

As can be seen in Figure 7, the MSD values are higher as expected in untreated cells, as compared to the same cells after ATP depletion. The slope of the MSD curves (α) in logarithmic scale is higher also before ATP depletion, relative to after ATP depletion. These results are in agreement with work by Guo et al. and others [1,7], in which intracellular, ATP-dependent forces were considered to augment MSD and *α* values in active cells, under normal condition. Figure 8 summarizes the results of *K_α_* and *α* in the 12 Jurkat cells before and after ATP depletion.

Both $K_{\alpha }$ and *α* are higher in untreated cells relative to the same cells after ATP depletion. The ATP-generated mechanical work in active cells may augment the random forces, which impact intracellular vesicles and structures and, by that, increase *K_α_*. These forces may also increase the power of the basal thermal diffusion, bounded by the elasticity of the medium. This medium, in turn, becomes an active and dynamic elastic gel. This increase in power is reflected in Equation (7) (Section 3.1) as high *α* values in active cells under normal conditions. 

We note that the range of appropriate thresholding values for object segmentation may result in variations of the MSD plots and in the resultant diffusion parameters of $K_{\alpha }\;\mathrm{and}\;\alpha$ (see Methods and Supplementary Note 2). Considering permissive variations of the threshold values within this range resulted in variations in $K_{\alpha }$ values with a standard-deviation (SD) of up to 0.00385 and in α of up to 0.151 (Figure S2). These values can be considered as the maximal (rather than typical) effective errors of the MSD analysis, and include cell-to-cell variability and limitations that are related to our diffraction-limited imaging and to its temporal resolution [34] (see methods).

**Figure 9:**
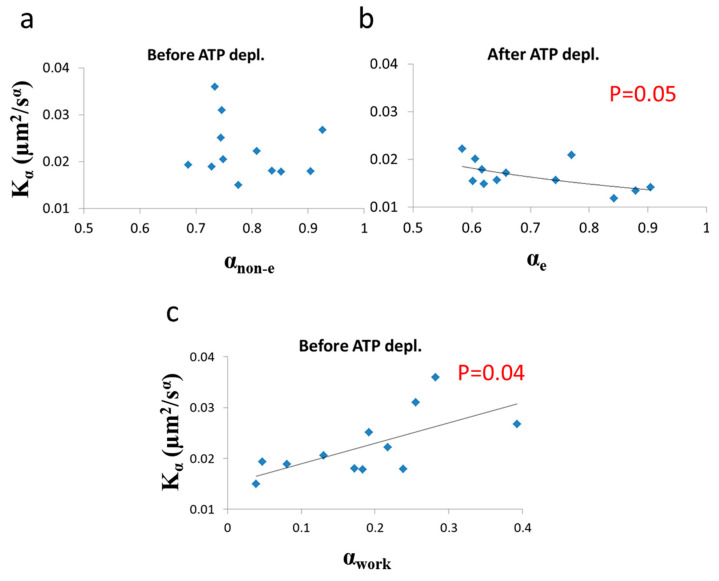
Correlation of diffusion characteristics before and after ATP depletion. (**a**) *α* (non-equilibrium condition) and $K_{\alpha }$ values in 12 Jurkat cells before ATP depletion; (**b**) *α* (equilibrium condition) and $K_{\alpha }$ values in the same 12 Jurkat cells after ATP depletion; (**c**) α_work_ and $K_{\alpha }$ values in the 12 Jurkat cells before cellular ATP depletion. α_work_ was calculated according to Equations (7) and (10) utilizing the exponential regression (of (**b**)) for calculating the expected α_e_ component (of α_non-e_) from $K_{\alpha }$ values. Regression *p*-values are depicted in (**b**) and (**c**) (no significant correlation was found in (**a**)).

Next we analyzed the correlation between $K_{\alpha }$ and *α* under each of the two cellular conditions (i.e., before and after ATP depletion). The correlation results are presented in Figure 9.

As predicted in the previous theoretical section (Section 3.1), a negative correlation between *α* and $K_{\alpha }$ was found in cells under thermodynamic equilibrium after ATP depletion. According to Equation (6), the correlation between *α* and $K_{\alpha }$ is negative, parametric and non-linear (Figure 5). The experimentally found correlation between *α* and $K_{\alpha }$ in ATP-depleted Jurkat cells (Figure 9b) matches this prediction. If the energy level of the system is not constant, the correlation between *α* and *K_α_*, suggested by formula 6, will no longer hold, since *α* and $K_{\alpha }$ will be influenced to a different extent by different amount of work done on the system (as was discussed previously). Furthermore, as proposed in Equation (7), α_non-e_ in non-equilibrium is the result of two influences: α_e_, which mainly reflects the elasticity of the medium, and α_work_, which reflects the contribution of intracellular mechanical work to *α*. An experimental regression formula for calculating α_e_ values from $K_{\alpha }$ values can be derived from the exponential relation between *α* and $K_{\alpha }$ in ATP-depleted cells (see Figure 9b):

(10) $$\alpha_{e}=0.53K_{\alpha }^{-0.46}$$

and used in non-equilibrium states. Using in this case the estimated α_e_ values determined by Equation (10) enables estimation of α_work_ values under the non-equilibrium conditions. This principle may be applied to active living cells, to relate α_work_ values to *K_α_* values (Figure 9c), showing the positive influence of cellular mechanical work on *K_α._*

### 3.3. Relation between Diffusion Characteristics and Intracellular Homogeneity

Cells have been shown to undergo an LLPS process that tends to segregate intracellular material into distinct areas, without the need for intracellular, membrane-bound organelles [14,15,16]. LLPS has been shown to influence cells’ physiology and pathophysiology. Under extreme conditions, a lack of sufficient LLPS has been related to malignancy [17,18]. In contrast, a too high extent of LLPS has been related to degenerative diseases [19,20,21,22,23]. Therefore, it is important to shed light on the biophysical mechanisms that may control it. 

Here, we hypothesized that the (global and relatively static) LLPS state of the cell is closely related to its dynamic characteristics (i.e., anomalous diffusion). To study this potential relation, we analyzed the correlation between the diffusion characteristics *α* and $K_{\alpha }$ and the intracellular LLPS state, both measured in the same cells. For that, we combined serial DIC microscopy images that were obtained as previously described and confocal microscopy images of nucleoli, which are influential membraneless LLPS organelles [14]. While nucleoli reside in the nucleus and not in the cytoplasm, their imaging here is advantageous, since the nucleus occupies in Jurkat cells most of the cell volume. Fibrillarin is a typical constituent of nucleoli and can be clearly imaged in cells that were transfected with GFP-tagged fibrillarin, as we did here [35,36]. The extent of nucleoli LLPS was measured by analyzing the Coefficient of Variation (CV) of pixels’ intensity in the GFP-fibrillarin images (further referred to as ‘fibrillarin intensity’). We provide an analysis of the effect of imaging noise on the CV of fibrillarin intensity in the SI (see Supplementary Note 3 and Figure S3). We conclude there that imaging noise does not significantly affect the CV of the imaged fibrillarin in our measurements. In these images, low CV values relate to more homogeneity of fibrillarin and low LLPS, while high CV values relate to the opposite. In that way, diffusion parameters and LLPS (via nucleoli morphology) were measured and correlated in multiple Jurkat T cells, each before and 30 min after ATP depletion (see Section 2.1.3 in Methods). 

**Figure 10:**
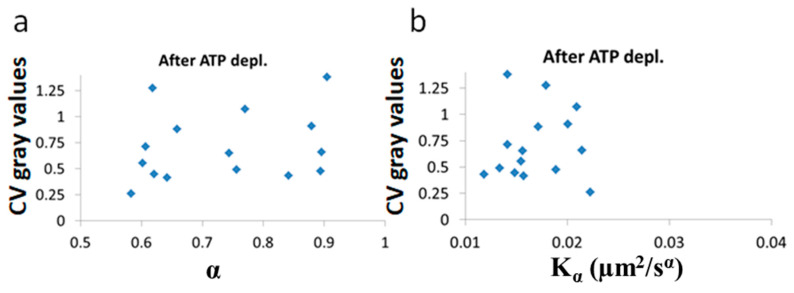
No correlation of LLPS and diffusion characteristics after ATP depletion. $K_{\alpha }$, α and Coefficient of Variation (CV) of fibrillarin images pixels intensities in 15 Jurkat cells after ATP depletion: (**a**) CV values vs $K_{\alpha }$ and (**b**) CV values vs α. CV values were calculated for all pixel intensities within the footprint of individual cells (N = 15).

**Figure 11:**
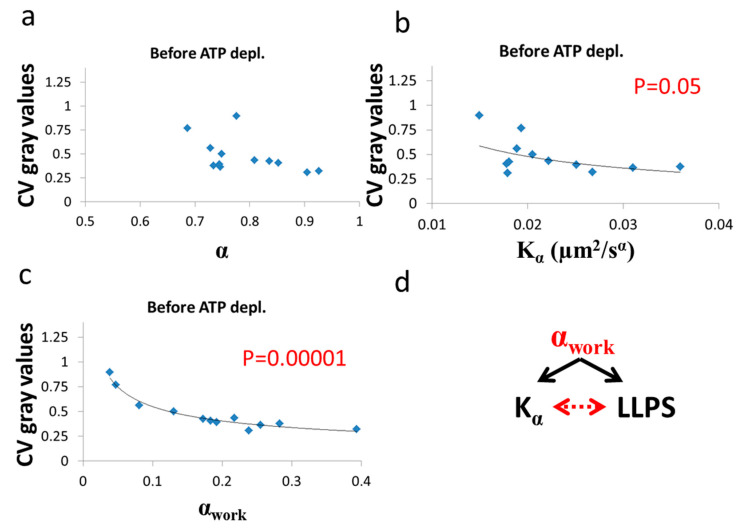
Correlation of LLPS and diffusion characteristics in untreated cells. $K_{\alpha }$, α and CV of fibrillarin intensities in 12 Jurkat cells before ATP depletion: (**a**) CV values vs α.; (**b**) CV values vs $K_{\alpha }$; (**c**) CV values vs α_work_. CV values were calculated for all pixel intensities within the footprint of individual cells (N = 12). *p*-values for the exponential regression are depicted in (**b**) and (**c**). No significant correlation was found in (**a**); (**d**) schematic representation of the impact of α_work_ on both $K_{\alpha }$ (Figure 9c) and LLPS (Figure 11c) that may create the correlation between $K_{\alpha }$ and LLPS (Figure 9b).

Next, we show the results of the CV of fibrillarin intensity vs either *α* or $K_{\alpha }$ after (Figure 10) and before (Figure 11) ATP depletion.

After ATP depletion, when no mechanical work is produced in the cells (equilibrium state), no correlation could be found between LLPS (highlighted by nucleoli) and the diffusion characteristics $K_{\alpha }$ and α (Figure 10a,b). In contrast, in active cells before ATP depletion, $K_{\alpha }$ and α_work_ negatively correlated with the CV of fibrillarin pixels intensities and LLPS (Figure 11b,c). Since such cells are in non-equilibrium and mechanical work is produced by the cells, LLPS is negatively influenced by α_work_. This means that when the mechanical work produced in the cell is high, its intracellular content becomes more homogenous and the LLPS state decreases.

Due to the positive correlation between α_work_ and $K_{\alpha }$ in active cells (Figure 9c), it is expected that the co-influence of α_work_ on $K_{\alpha }$ and on LLPS will further create a negative correlation between $K_{\alpha }$ and LLPS, as shown in Figure 11b.

### 3.4. Evaluation of Entropy in Jurkat Cells before and after ATP Depletion and Its Relation to the Cells LLPS

**Figure 12:**
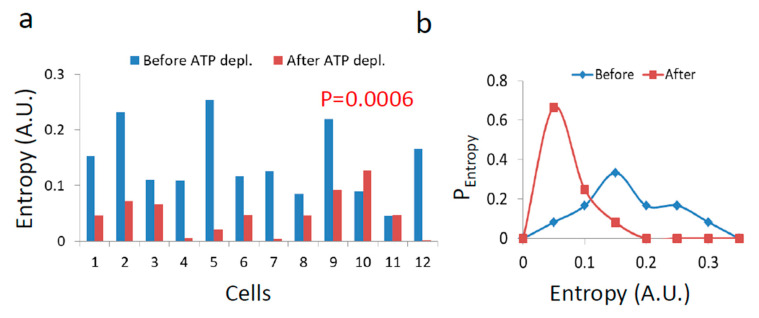
Entropy in Jurkat cells before and after ATP depletion. (**a**) Entropy values (according to Equation (2)) in each cell before and after ATP depletion (N = 12); (**b**) Histogram of entropy values before and after ATP depletion.

The resulting diffusion parameters before and after ATP depletion (Figure 7 and Figure 8) predict that the generalized entropy according to Equation (2) will be higher in the active cells than in cells after ATP depletion. The results that are summarized in Figure 12 confirm this prediction. 

Untreated cells are expected to have high entropy values due to intracellular mechanical work. Following the negative correlation between LLPS and non-equilibrium diffusion parameters (α_work_ and $K_{\alpha }$ in Figure 11b,c), it is further expected that the entropy for such cells will also be negatively correlated with the LLPS state. In contrast, the absence of correlations between equilibrium diffusion parameters and LLPS in ATP-depleted cells (in Figure 10) suggests that entropy values would also have no correlation to LLPS after ATP depletion.

**Figure 13:**
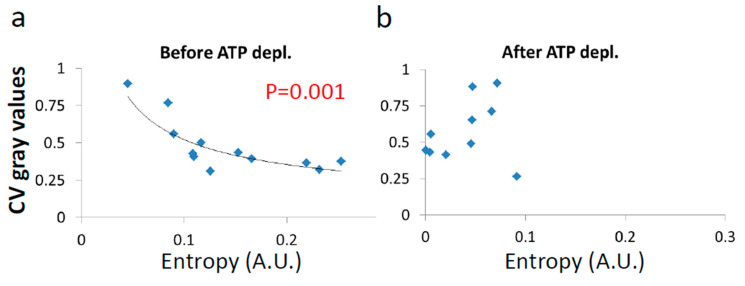
Correlation between the CV of fibrillarin intensities and entropy in cells (**a**) before, and (**b**) after ATP depletion. CV was calculated for all pixel intensities within the footprint of individual cells (N = 12). *p*-values for the exponential regression are depicted in (**a**).

The correlation results between cellular entropy and LLPS (expressed by the CV of fibrillarin intensities) are presented in Figure 13. 

As expected, we find that entropy of untreated cells negatively correlates to their medium LLPS (Figure 13a), facilitating homogeneity of their content. In contrast, after ATP depletion entropy is no longer correlated with this homogeneity and LLPS (Figure 13b). The extent of organization of the cell content is thermodynamically balanced by the counter action of entropy against the chemical and potential energy attraction. In physiologically-paralyzed ATP-depleted cells, entropy is driven only by thermal agitation forces that are too small to balance the attraction forces of the cell constituents. This tends to create dominance of assembly processes and over compartmentation of the intracellular content. On the other hand, in active cells, constant mechanical work contributes to entropy and counter-acts and dominates the attraction forces and potential energy. This work produces mechanical forces that enable entropy to govern and increase the homogeneity of the cell content. By that, the cell could become even more flexible and functionally active.

## 4. Discussion

Here we measured and analyzed the dynamics of intracellular objects that take place under non-equilibrium conditions and are influenced by mechanical work inside cells. By analyzing these dynamics as diffusion-like, we were able to obtain theoretical relations between the power of the diffusion (α) and the diffusion coefficient ($K_{\alpha }$); and to identify the total energy and the generalized entropy of the system. Under these conditions, we could further theoretically relate the generalized entropy and the total energy to the diffusion characteristics, $K_{\alpha }$ and α.

Considering diffusion in the intracellular medium when no work is added, as under equilibrium condition, enables characterization of a theoretical relation between $K_{\alpha }$ and α. This relation is not expected to hold under non-equilibrium intracellular conditions when work is produced by or applied to the cellular system. Then, $K_{\alpha }$ and α values (of the equilibrium state) are expected to increase to a different extent for each different energy level. While work is added to the system, which in turn changes from equilibrium to non-equilibrium, the increase in *α* (namely, $∆\alpha =\alpha_{work}$) can be considered as a linear addition to the basic equilibrium *α* value ($\alpha_{e}$). Therefore, the total *α* of the non-equilibrium state ($\alpha_{non-e}$) is composed of these two components: $\alpha_{e}$ and $\alpha_{work}$. Accordingly, entropy in living cells can also be regarded as composed of two components: An equilibrium (thermal) entropy component, and a non-equilibrium component due to ATP-dependent mechanical work. As a result, the generalized entropy of live cells (in non-equilibrium) is expected to be larger than of paralyzed ATP-depleted cells (in equilibrium). This enables live cells to have efficient intracellular motion and flexibility. Too much intracellular entropy carries the risk of irreversible disruption of intracellular order and malignant transformation [37,38]. The logarithmic relation between mechanical energy in the cell and its entropy aids to restrict the possibility of a steep rise in entropy levels due to excess of mechanical energy. The cell can control the equilibrium component of entropy by increasing its elasticity, which may also aid to reduce its total entropy. 

We experimentally compared the diffusion characteristics of intracellular objects (mostly vesicles) in active Jurkat cells (non-equilibrium condition) and after their ATP depletion (i.e., in the same cells, now under equilibrium condition). The Jurkat cells are non-adherent and thus allow the study of cellular mechanical fluctuations and their effect on content dynamics and thermodynamics. Otherwise, the attached surrounding of adherent cells is capable of delivering forces and work to the cells under study, which would have interfered with the related measurements.

This comparison between active and ATP-depleted cells supported our theoretical predictions. $K_{\alpha }$ and α were higher in active non-treated cells, as compared to the same cells after ATP depletion. $K_{\alpha }$ and α negatively correlated in ATP-depleted cells (equilibrium condition) while no correlation could be identified in active non-treated cells (non-equilibrium condition). In the active cells, $\alpha_{work}$ positively correlated with $K_{\alpha }$, as the extent of work that was added to the non-equilibrium system (and relates to $\alpha_{work}$) is expected not only to increase the total *α* ($\alpha_{non-e}$), but also to increase $K_{\alpha }$.

Intracellular LLPS state of intracellular content is a crucial biophysical parameter for cell functioning, and relates to many pathophysiology conditions, including malignancy and degenerative diseases. Cellular LLPS (demonstrated by nucleoli morphology) was found to negatively correlate with $\alpha_{work}$, and probably as a consequence of that, also to $K_{\alpha }$ in active cells. No correlation to intracellular diffusion characteristics could be found in non-active, ATP-depleted cells.

As expected, the generalized entropy values, according to the theoretical Equations (2) and (8), were higher in active untreated cells, as compared to the same cells after ATP depletion. The level of cellular entropy related to cellular LLPS only in active cells. Therefore, we conclude that the non-equilibrium contribution to the entropy is essential to enable the cell control over its content homogeneity and LLPS state. These demonstrated relations between intracellular mechanical work fluctuations and diffusivity that facilitate cellular content entropy and homogeneity highlight an important biophysical mechanism to control cell physiology. 

## 5. Conclusions

Active cells can be regarded as consisting of a complex, non-homogenized, multi-component, viscoelastic gel in a non-equilibrium steady-state. Such a system is hard to study via classical physics and state-of-the-art non-equilibrium statistical physics. Here, our use of simplifying yet cautious assumptions of steady-state enable an extended use of equilibrium thermodynamic state functions to characterize live cells. Specifically, we determine relations between dynamic and easily measurable parameters as diffusion characteristics and thermodynamic state functions, including energy and entropy. Arguably, linking between the dynamic diffusion parameters (mainly the power of diffusion), physical state functions of cells (mainly generalized entropy), and physiologically-related biophysical parameters, such as LLPS, is important for our understanding of cardinal processes in cells and, ultimately, our ability to influence them in a controlled fashion.

## Supplementary Materials

The following are available online at https://www.mdpi.com/1099-4300/21/10/962/s1, Supplementary Note 1: Distribution of Tracked Particles Diameter in the Measured Cells, Supplementary Note 2: $K_{\alpha }$ and α Values as a Function of the Width of the Threshold Window in DIC Analyses, Supplementary Note 3: The Effect of Imaging Noise on the Captured Fibrillarin Distribution, Figure S1. The distribution of the estimated diameter of the tracked particles in the group of cells (N = 12): before and after ATP depletion, Figure 2. $K_{\alpha }$ and α values as a function of the width of the threshold window in DIC analyses. Results are shown for the representative cell, shown in Figure 1, Figure S3. CV of fibrillarin intensities in images of 12 Jurkat cells before and after ATP depletion. CV_Noise_ results of each cell and condition are presented as error bars on top of CV_fibrillarin_.
