# Supplementary material for: ATP-Dependent Diffusion Entropy and Homogeneity in Living Cells
Source: Entropy (Basel). 2019 Oct 1;21(10):962. doi: 10.3390/e21100962 (PMC7514293; doi:10.3390/e21100962)
Supplement: Supplementary file 1 [file entropy-21-00962-s001.pdf]

Supplementary Materials

Supplementary Note 1: Distribution of Tracked Particles Diameter in the Measured Cells

The analyzed distributions of particles diameter are represented in Figure S1 for the two metabolic conditions: non-treated cells and the same cells after ATP depletion.

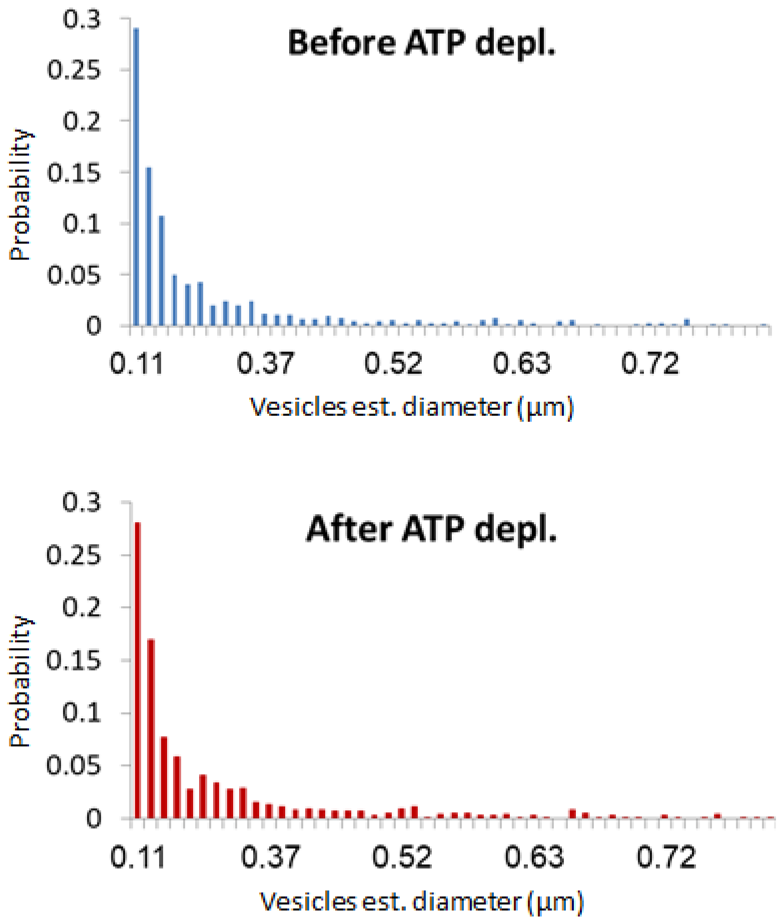

Figure S1. The distribution of the estimated diameter of the tracked particles in the group of cells (N=12): before and after ATP depletion. .

As can be seen in Figure S1, most of the particles are in the size range of intracellular vesicles or organelle (0.1 to ~1μm).

Supplementary Note 2: K_α and α Values as a Function of the Width of the Threshold Window in DIC Analyses

Object segmentation in DIC images (and image stacks) requires thresholding. The thresholding is performed by choosing a range of gray levels (i.e. a window). Thresholding results in a binary image where the objects under study can be readily segmented. Typically, a suitable value that defines the window edge is identified by the ImageJ thresholding algorithm. Still, the choice of the window size is user-defined (i.e. subjective) and is aimed at optimizing the appearance of the objects under study. Thus, thresholding may affect the experimental results, primarily K_α and α. To quantify such thresholding effects, we studied a range of window widths and calculated the resultant variations in the K_α and α. Results are shown for the representative cell, shown in Figure 1.

We first calculated K_α results for a range of window sizes between 0 and 20 (Figure S2 upper panel). For this cell, our user-defined choice was a window size of 9. The SD of the results of permissive window sizes between 6 and 12 (see black lines in Figure S2) was 0.00220. For comparison, when we applied our user-defined window size for thresholding of individual cells, the SD of K_α for the entire set of (12) imaged cells was 0.00316. Thus, the effective error of choosing permissive window sizes would be 0.00385 (as compared to 0.00316, when applying the user-defined thresholds).

Following the same procedure, we assessed the effect of the window size for thresholding on the α values (Figure S2 lower panel). The SD of the results of permissive window sizes between 6 and 12 (see black lines in Figure S1) was 0.0959. In contrast, the SD of α values for the entire set of imaged cells was 0.117. Thus, the effective error of choosing permissive window sizes would be 0.151.

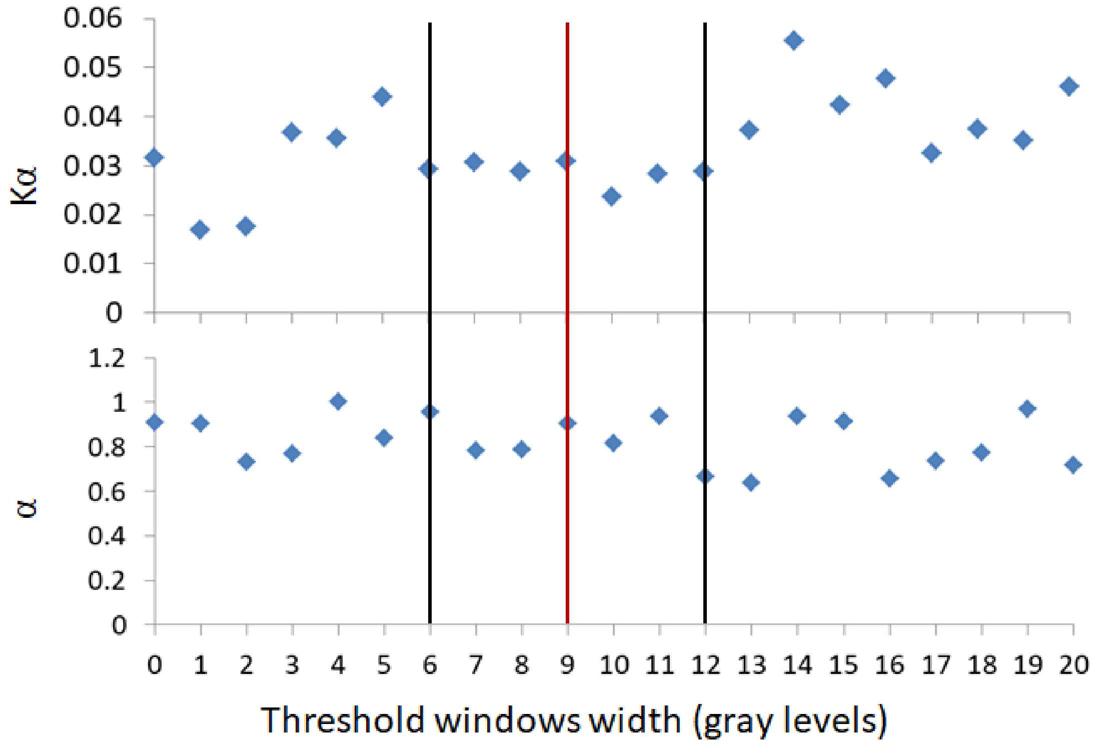

Figure 2. K_α and α values as a function of the width of the threshold window in DIC analyses. Results are shown for the representative cell, shown in Figure 1.

Supplementary Note 3: The Effect of Imaging Noise on the Captured Fibrillarin Distribution

To assess the effect of imaging noise on the results of the CV of fibrillarin, we evaluated the possible contribution of such noise to the CV result. We considered the Noise to be intensity fluctuations in pixels from image areas where the signal should be zero. In our setup the extracellular areas should not have significant concentration of fibrillarin, which is expressed inside the cells and is not expected to pass the cell membrane. The SD of intensity fluctuations in such pixels was calculated for each cell and each condition (before and after ATP depletion). Assuming additively of noise, those SD results were divided with the average fibrillarin intensity. This is in accordance with the division of the SD of intracellular pixels having fibrillarin intensities, with the average fibrillarin intensity inside the cell, which produces the $CV_{fibrillarin}$ results. Thus, $CV_{Noise} = \frac{SD_{Noise}}{<i_{fibrillarin}>}$, and it is already included in our measured $CV_{fibrillarin}$. We show $CV_{fibrillarin}$ and CV_{Noise} in Figure S3 below for all imaged cells before and after ATP depletion. As can be seen, the evaluated level of the imaging noise is relatively small and does not significantly affect the CV of the imaged fibrillarin in our measurements.

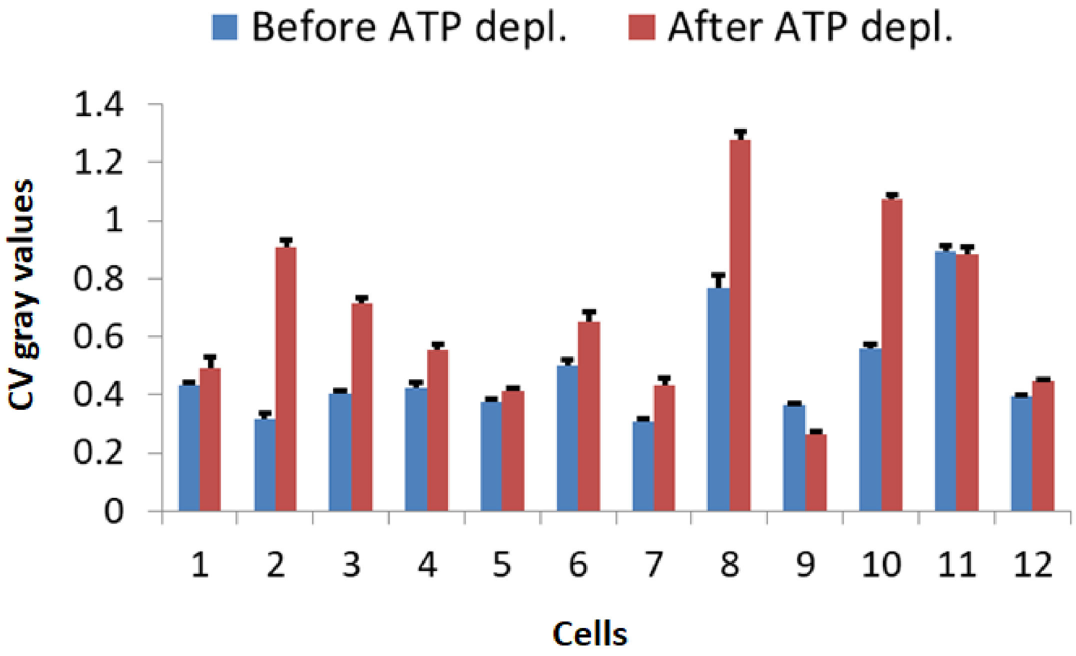

Figure S3. CV of fibrillarin intensities in images of 12 Jurkat cells before and after ATP depletion. CV_{Noise} results of each cell and condition are presented as error bars on top of $CV_{fibrillarin}$.
